# Transforming body composition with semaglutide in adults with obesity and type 2 diabetes mellitus

**DOI:** 10.3389/fendo.2024.1386542

**Published:** 2024-06-04

**Authors:** Beatriz Rodríguez Jiménez, Pablo Rodríguez de Vera Gómez, Samuel Belmonte Lomas, Ángel Manuel Mesa Díaz, Irene Caballero Mateos, Irene Galán, Cristóbal Morales Portillo, María Asunción Martínez-Brocca

**Affiliations:** Servicio de Endocrinología y Nutrición, Hospital Universitario Virgen Macarena, Seville, Spain

**Keywords:** obesity, type 2 diabetes mellitus, body composition, phase angle, fat mass, lean body mass, visceral fat area, HbA1c

## Abstract

**Background:**

Glucagon-like peptide-1 receptor-agonists (GLP-1ra), such as semaglutide, have emerged as promising treatments, demonstrating sustained weight reduction and metabolic benefits. This study aims to assess the impact of oral and subcutaneous semaglutide on body composition and metabolic parameters in patients with T2DM and obesity.

**Methods:**

A 24-week quasi-experimental retrospective study including adults with T2DM and obesity (BMI ≥ 30 kg/m²) who were treated with either daily-oral or weekly-subcutaneous semaglutide. Body composition was measured using bioelectrical impedance analysis, evaluating fat mass, fat-free mass, total body water, skeletal muscle mass, and whole-body phase angle. Analytical parameters included lipid profile and glycaemic control. Statistical analyses were performed using SPSS v.26.

**Results:**

Participants (n=88) experienced significant weight loss after treatment with semaglutide (9.5% in subcutaneous, 9.4% in oral, P<0.001). Weight reduction primarily resulted from fat mass reduction without substantial lean mass compromise. Visceral fat area decreased, whiles phase-angle remained stable. Improvements in lipid profiles and glycaemic control were observed, with a decrease in both HbA1c and insulin requirements. Multivariate analysis demonstrated comparable impacts of oral and subcutaneous semaglutide on body composition.

**Conclusion:**

Semaglutide, administered orally or subcutaneously, demonstrated positive effects on body composition, metabolic and glycaemic control in patients with T2DM and obesity. This real-world study highlights the potential of bioelectrical impedance analysis in assessing antidiabetic drugs’ impact on body composition, providing valuable insights for future research and clinical applications.

## Introduction

Treating type 2 diabetes mellitus (T2DM) with the aim of avoiding its well-known long-term complications has been a therapeutic challenge over the last century. Increasing knowledge of hormones within the incretin family, has led to the identification and development of a series of agents that expand therapeutic options when treating patients with these pathologies. In particular, glucagon-like peptide-1 (GLP-1) receptor agonist (GLP-1ra) has demonstrated sustained, clinically significant body weight reductions in patients with T2DM and obesity (1–3).

GLP-1 is a gut hormone secreted by distal enteroendocrine L-cells, located in the small bowel and colon (4). It is released in response to meal ingestion, stimulating glucose-dependent insulin secretion by pancreatic beta-cells (5). Additionally, glucagon secretion is suppressed by GLP-1 (4), leading to a glucose-dependent reduction of hepatic glucose production (5). This process is regulated by glucose levels, mitigating the risk of hypoglycaemia (6). GLP-1 receptors are widely expressed in tissues beyond the gastrointestinal tract, including the endocrine pancreas, kidneys, cardiovascular system, and various regions of the nervous system (4, 5, 7). Considering the broad expression of GLP-1 receptors throughout the body, GLP-1 serves multiple roles beyond glucose homeostasis. For instance, gastric emptying rate is decelerated in response to glucose increase. Modulation of satiety is also mediated by GLP-1 (8), as well as regulation of energy intake via hypothalamic feeding circuits (9, 10).

GLP-1ra mimic native GLP-1’s pancreatic actions, stimulating weight loss and improving glycaemic control (5), as well as promoting physiological effects such as cardiovascular risk reduction (5, 11, 12), atherogenesis prevention through vascular inflammation modulation (13, 14) and renoprotective and neuroprotective effects (15–18), which enhance GLP-1ra’s beneficial outcomes. This provides medical professionals a promising tool to treat patients living with obesity and T2DM, beyond insulin and prior diabetic management approaches. However, GLP-1 based treatments can also entail adverse effects, which are mainly gastrointestinal, notably nausea, vomiting, and diarrhoea (19). Such effects usually decrease over time with continued therapy and can be mitigated by adjusting the dosage (20).

Semaglutide is a GLP1-ra, with 94% amino acid sequence homology with native GLP-1 (21). Structural modifications of the molecule have enabled semaglutide to be less susceptible to degradation by dipeptidyl peptidase-4, thus extending its half-life to nearly a week (22). It is already known that semaglutide achieves reduction in body weight which in turn improves glycaemic control and thus reduces risk associated with poorly controlled T2DM (23), but it is still necessary to expand the information regarding its effect on body composition, which is why we performed this investigation.

Bioelectrical impedance (BIA) represents a non-invasive, relatively fast and simple tool to estimate body composition, providing information beyond body mass index (BMI) and other anthropometric measurements.

Within this study, we aim to assess the impact of GLP-1ra Semaglutide on body composition of patients with T2DM and obesity, as a reflection of their nutritional status, while evaluating possible metabolic changes after a 24-week period of treatment.

## Materials and methods

### Design and subjects

We conducted a 24-week follow-up quasi-experimental retrospective study to assess changes in body composition and the effect of semaglutide on analytical parameters. Recruitment was carried out systematically and consecutively in the Endocrinology and Nutrition Department of our hospital, the Virgen Macarena University Hospital in Seville, during the years 2020–2022.

Adults (18 years or older) with T2DM, insufficient glycaemic control [glycated haemoglobin (HbA1c) ≥ 7%] and Obesity (BMI ≥30kg/m^2^) who underwent treatment with GLP1-ra, both in its subcutaneous and oral forms, were included in our study. Those who were already taking GLP1-ra or had in the past, subjects with a follow-up period shorter than 12 months and those who had to interrupt treatment due to adverse events or drug intolerance (predominantly gastrointestinal symptoms), were excluded from the study.

Subjects were divided into 2 groups depending on the intended route of Semaglutide administration they were to receive: daily oral semaglutide (OS) or weekly subcutaneous semaglutide (SS). Due to significant differences in their baseline characteristics, both groups were independently analysed within the context of the study, which was conducted under real-life conditions (see **Tables 1**, **2**, where differences in baseline characteristics between treatment groups are shown with *).

**Table 1:** Baseline characteristics of the sample divided into treatment groups.

	SUBCUTANEOUS SEMAGLUTIDE (n = 55)	ORAL SEMAGLUTIDE (n = 33)
Age (years)*	55.3 (10.4)	61.8 (7)
Gender		
*Male*	29 (52.7%)	22 (66.7%)
*Female*	26 (47.3%)	11 (33.3%)
BMI*	40.1 (11)	33.2 (3.9)
**Years with DM**	7.2 (6.3)	9.6 (6.3)
**Age at DM diagnosis (years)**	50.4 (10.9)	53.6 (6.5)
**Severe hypoglycaemia events**	0 (0.0%)	1 (3%)
Diabetic retinopathy*	0 (0.0%)	3 (9.1%)
**Hypertension**	42 (76.4%)	26 (78.8%)
**Dyslipidemia**	39 (70.9%)	26 (78.8%)
**Chronic Kidney disease**	3 (5.5%)	6 (18%)
**OSAS**	9 (16.4%)	9 (27.3%)
Ischemic heart disease*	0 (0.0%)	6 (18.2%)
Heart failure*	1 (1.8%)	5 (15.2%)
**Stroke**	0 (0.0%)	1 (3%)
**NAFLD**	10 (18.2%)	9 (27.3%)
**Smoker**	10 (18.2%)	8 (24.2%)
Alcohol*	1 (1.8%)	4 (12.1%)
**Metformin**	39 (70.9%)	25 (75.8%)
**iSGLT2**	20 (36.4%)	15 (45.5%)
**Sulfonylureas**	8 (14.5%)	6 (18%)
**DPP-4 inhibitors**	10 (18.2%)	9 (27.3%)
**Basal insulin**	24 (43.69%)	17 (51.5%)
**Prandial insulin**	10 (18.2%)	7 (21.2%)
**ACE inhibitors**	39 (70.9%)	20 (60.6%)
β-Blockers*	9 (16.4%)	11 (33.3%)
**α-Blockers**	1 (1.8%)	2 (6.1%)
**Diuretics**	22 (40%)	14 (42.4%)
**Calcium channel blockers**	9 (16.4%)	5 (15.2%)
**Spironolactone**	2 (3.6%)	3 (9.1%)
Statin therapy:		
*None*	29 (52.7%)	13 (39.4%)
*Low intensity*	3 (5.5%)	1 (3%)
*Moderate intensity*	17 (30.9%)	10 (30.3%)
*High intensity*	6 (10.9%)	9 (27.3%)
**Ezetimibe**	6 (10.9%)	4 (12.1%)
**Fibrates**	5 (9.16 %)	7 (21.2%)
**Anticoagulants**	4 (7.3%)	2 (6.1%)
**Antiaggregant therapy**	9 (16.4%)	11 (33.3%)

*Baseline characteristics in which we found significant differences (p < 0.05) between treatment groups.

DM, Diabetes Mellitus; OSAS, Obstructive sleep apnea syndrome; NAFLD, Nonalcoholic fatty liver disease; iSGLT2i, sodium-glucose linked transporter inhibitors; DPP-4, Dipeptidyl peptidase-4; β-Blockers, beta-blocker or β-adrenoreceptor antagonists; α-Blockers, alpha blockers or α-adrenoreceptor antagonists.

**Table 2:** Variation in body composition parameters from baseline to end of follow-up (6 months) using bioelectrical impedance analysis.

	SUBCUTANEOUS SEMAGLUTIDE (n=55)				ORAL SEMAGLUTIDE (n = 33)			
	BASELINE mean (SD)	6 MONTHS mean (SD)	DIFFERENCE [CI 95%]	P-value^a^	BASELINE mean (SD)	6 MONTHS mean (SD)	DIFFERENCE [CI 95%]	P-value^a^
Weight (kg)*	109.2 (24.6)	98.5 (21.6)	-10 [-11.9; -8.2]	<0.001	94.8 (15.6)	86.2 (16.3)	-8.6 [-10.3; -6.8]	<0.001
BMI (kg/m2)*	40.1 (11)	36.1 (9.3)	-3.7 [-4.4; -3]	<0.001	34.2 (3.9)	31 (4.2)	-3.1 [-3.7; -2.5]	<0.001
Body Fat Mass (kg)*	50.5 (17.6)	41.3 (15)	-8.5 [-10.2; -6.9]	<0.001	39.9 (8)	31.9 (8.7)	-8 [-9.7; -6.2]	<0.001
**Soft Lean Mass (kg)**	56.4 (13.6)	54.1 (12.2)	-1.7 [-2.4; -1]	<0.001	51.9 (10.3)	51.2 (10.2)	-0.7 [-1.5; 0.2]	0.112
Fat Free Mass (kg)*	59.3 (13.5)	57.1 (12.7)	-1.7 [-2.5; -0.9]	<0.001	54.9 (10.9)	54.3 (10.8)	-0.6 [-1.5; 0.3]	0.162
**Fat mass (%)**	45.7 (9.3)	41.4 (9.1)	-4 [-4.9; -3.1]	<0.001	42.2 (5.6)	36.8 (6.1)	-5.2 [-6.7; -3.6]	<0.001
**Fat free mass (%)**	54.9 (9.2)	58.6 (9.1)	3.6 [2.8; 4.6]	<0.001	57.8 (5.6)	63.2 (6.1)	5.2 [3.6; 6.7]	<0.001
Fat Free Mass Index (kg/m2)*	22.1 (10.1)	21.1 (7.7)	-0.6 [-0.9; -0.4]	<0.001	19.7 (2.2)	19.5 (2.3)	-0.2 [-0.5; 0.1]	0.197
**Fat Mass Index (kg/m2)**	18.2 (6.4)	15.1 (5.6)	-3.1 [-3.7; -2.5]	<0.001	14.5 (3)	11.5 (3.1)	-3 [-3.7; -2.3]	<0.001
Visceral Fat Area (cm2)*	227.1 (62.2)	196.5 (54.4)	-30.2 [-37.5; -23.2]	<0.001	203.6 (37.1)	161.3 (47.3)	-42.3 [-52.9; -31.8]	<0.001
**Body Cell Mass (kg)**	38.6 (9.4)	37 (8.4)	-1.2 [-1.7; -0.7]	<0.001	35.5 (7.2)	35 (7.1)	-0.5 [-1.1; 0.1]	0.077
**Skeletal Muscle Mass (kg)**	33.1 (8.5)	31.7 (7.7)	-1.1 [-1.5; -0.6]	<0.001	30.3 (6.5)	29.9 (6.4)	-0.5 [-1; 0.1]	0.081
**Skeletal Muscle Index (kg/m2)**	8.6 (8.4)	8.3 (1.3)	-0.4 [-0.5; -0.2]	<0.001	8.1 (1.2)	7.9 (1.2)	-0.2 [-0.4; -0.1]	0.001
**Basal Metabolic Rate (Kcal)**	1651.3(292.1)	1604.3(274.5)	-36.3 [-53; -19.5]	<0.001	1555.7 (234.8)	1542.2(232.9)	-13.5 [-33; 5.9]	0.166
**Bone Mineral Content (kg)**	3 (1.5)	3.1 (1)	0.03 [0; 0.1]	0.237	3 (0.6)	3.1 (0.6)	0.1 [0; 0.1]	0.06
**Total body water (L)**	44 (10.6)	42.2 (9.5)	-1.3 [-1.9; -0.8]	<0.001	40.5 (8)	40 (8)	-0.5 [-1.2; 0.1]	0.117
**Intracellular Water (L)**	26.9 (6.5)	25.8 (5.9)	-0.8 [-1.2; -0.5]	<0.001	24.8 (5)	24.4 (4.9)	-0.4 [-0.8; 0]	0.078
**Extracellular Water (L)**	16.2 (3.4)	16.4 (3.7)	-0.2 [-0.9; 0.8]	0.684	16 (2.9)	15.6 (3.1)	-0.4 [-1.8; 0.9]	0.525
**50kHz Whole Body Phase** **Angle (°)**	5.1 (0.6)	5.1 (0.7)	-0.03 [-0.1; 0.1]	0.529	5 (0.8)	4.9 (0.7)	-0.1 [-0.2; 0]	0.068

*Parameters in which we found significant differences (p < 0.05) between treatment groups.

^a^ Univariate analysis using Wilcoxon test for paired data.

SD, Standard Deviation; CI, Confidence Interval; BMI, Body Mass Index.

**Figure 1:**
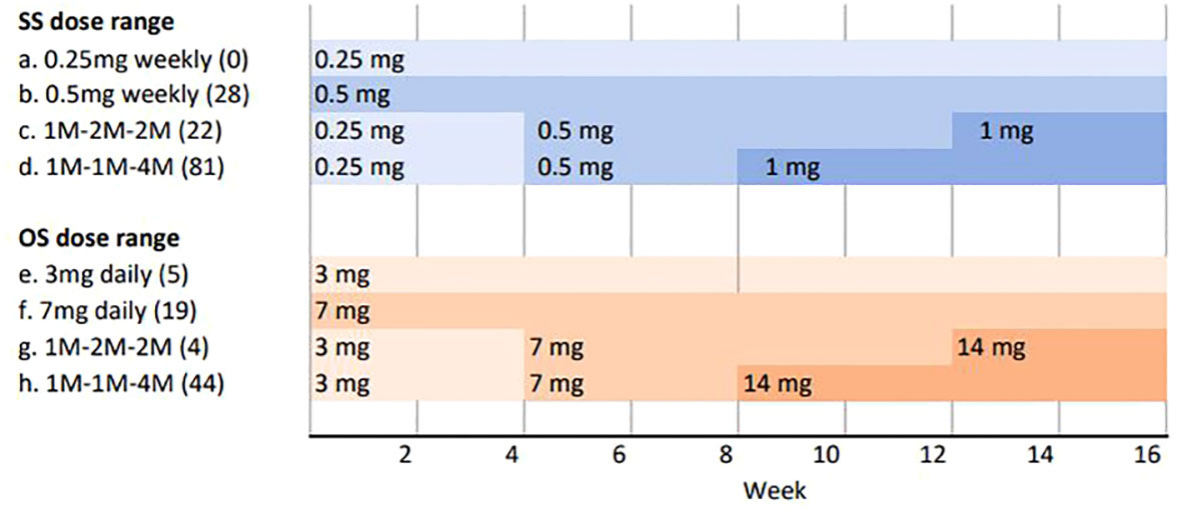
Study design–dose range administration. In order to obtain comparable data, only patients who followed a monthly dose escalation as represented by d and h, were selected. In brackets are the number of patients who followed each treatment scheme. SS, subcutaneous semaglutide; OS, oral semaglutide; mg, milligrams; M, months.

To ensure intervention homogeneity, we included patients who, based on their tolerance, adhered to the subsequent therapeutic regimen: following a 4-week dose escalation, medication was administered with a starting dose of 0.25mg once weekly, followed by 0.5mg weekly and finally 1 mg maintenance dose in SS; the initial dosage in the OS group was 3 mg taken daily for 4 weeks, followed by an additional 4 weeks at 7mg, and finally maintenance dose of oral semaglutide, 14 mg. Participants who were unable to adhere to this therapeutic protocol due to intolerance, adverse effects, or poor treatment compliance were excluded from the study (**Figure 1**). The remaining concomitant treatment (including hypoglycemic and hypolipidemic treatment) was indicated according to standard clinical practice guidelines.

Participants who experienced adverse effects or drug intolerance (mainly gastrointestinal symptoms including nausea, diarrhoea, and abdominal discomfort), rendering them unable to complete the prescribed treatment regimen, were excluded from bioimpedance analysis and subsequently not included in our study.

### Educational intervention

Patients were given general advice for incorporating physical activity and adopting a healthy lifestyle, based on the Mediterranean diet and active living to discourage sedentary behaviour. These guidelines were initially conveyed during the first visit and further emphasized by our nursing team in a brief 15-minute session, in which patients were instructed on the administration of semaglutide. Regarding dietary habits, patients received recommendations advocating for the consumption of fruits and vegetables, minimally processed foods, legumes, fish, and whole grains, along with hydration based on water over soft drinks and alcohol. Additionally, general physical activity guidelines were provided, encouraging an active lifestyle, avoiding sedentary behaviour, and recommending 30–60 minutes of daily aerobic exercise and 2–3 days per week of strength training, adapted to individual physical capabilities. This educational intervention was carried out as part of daily clinical practice, not with the aim of evaluating its impact.

### BIA measurement and analytical parameters

Body composition was measured using multi-frequency BIA (MF-BIA) (Inbody 770, Inbody Co., Ltd., Korea). All measurements were performed in the morning, at baseline and at the end of follow-up. Parameters including fat mass (FM), fat free mass (FFM), total body water, skeletal muscle mass and phase angle (PhA) where registered.

Blood tests were performed after 8 to 10 hours of fasting; samples were collected to measure analytical parameters such as haemoglobin, platelet count, fasting plasma glucose and HbA1c, creatinine, total cholesterol, and its lipoproteins.

### Study endpoints and assessments

The main study variable was reduction in body FM from baseline to week 24 of semaglutide treatment. Secondary efficacy end points at week 24 included changes in body composition, such as body water, skeletal muscle, and PhA; as well as modifications in analytical parameters; particularly lipid profile and glycaemic control.

### Statistical analysis

We estimated the required sample size to be 60 participants (30 for oral and 30 for subcutaneous semaglutide), assuming a two-sided hypothesis test with a 95% confidence level and accounting for a potential 10% loss. This estimation was based on a minimum clinically relevant difference of -10% in fat body mass at the conclusion of the follow-up (22, 23).

Categorical variables were expressed in numbers and percentages. Continuous variables were expressed as mean and standard deviation (SD) for normal distributions or median and interquartile range (IQ) if distributions were skewed. We used t-student test and Wilcoxon test for the comparison of continuous variables, normally distributed and nonnormally distributed respectively. Normality of quantitative data was tested using the Kolmogorov-Smirnov test. Homoscedasticity was evaluated using Levene´s test.

We conducted a multivariate analysis using multiple linear regression to adjust for predictor variables of FM loss percentage (dependent variable). Independent variables were selected from those that exhibited a significance level of p < 0.15 in the univariate analysis (data not shown) and those where significant differences were observed between treatment groups. The final number of included variables was determined based on the criteria of greater parsimony and better discrimination capacity, with reference to an area under the curve (AUC) >0.75 in the ROC curve analysis. The model’s goodness of fit was assessed using Hosmer-Lemeshow’s test.

The statistical significance was set at p < 0.05 (two-tailed hypothesis testing). The strength of association was presented as a mean difference and 95% confidence interval (CI).

Statistical analyses were performed using the SPSS v.26/PC software package (IBM Statistics).

### Ethics statement

Written informed consent was obtained from all subjects. This study was conducted in accordance with the Helsinki Declaration of 1975, as revised in 2008; and was approved by the Research Ethics Committee of the Hospital Universitario Virgen Macarena of Seville.

## Results

### Patients, baseline characteristics

A total of 203 patients were initially identified as potential candidates to participate in this study, of whom 88 were selected; 55 patients in the SS wing and 33 patients who received OS.

The mean age in the SS group was 55.3 ± 10.4 years, of which 47.3% were women, and had a mean BMI of 40.1 ± 11 kg/m2. In the OS group, mean age was 61.8 ± 7 years, 33.3% women and BMI 34.2 ± 3.9kg/m2. Disease duration was 7.2 ± 6.3 years for SS and 9.6 ± 6.3 years for OS.

Regarding the hypoglycaemic treatment our patients had been taking prior to the introduction of Semaglutide; a total of 64 patients were taking metformin [39 (70.9%) in the SS group and 25 (75.8%) in the OS group], and basal insulin was part of the glucose-lowering treatment of 41 subjects [24 (43.69%) in the SS group and 17 (51.5%) in the OS group]. Their clinical characteristics, comorbidities, and treatment they were receiving prior to the introduction of Semaglutide are listed in **Table 1**.

### Impact of semaglutide in body composition parameters

**Figure 2:**
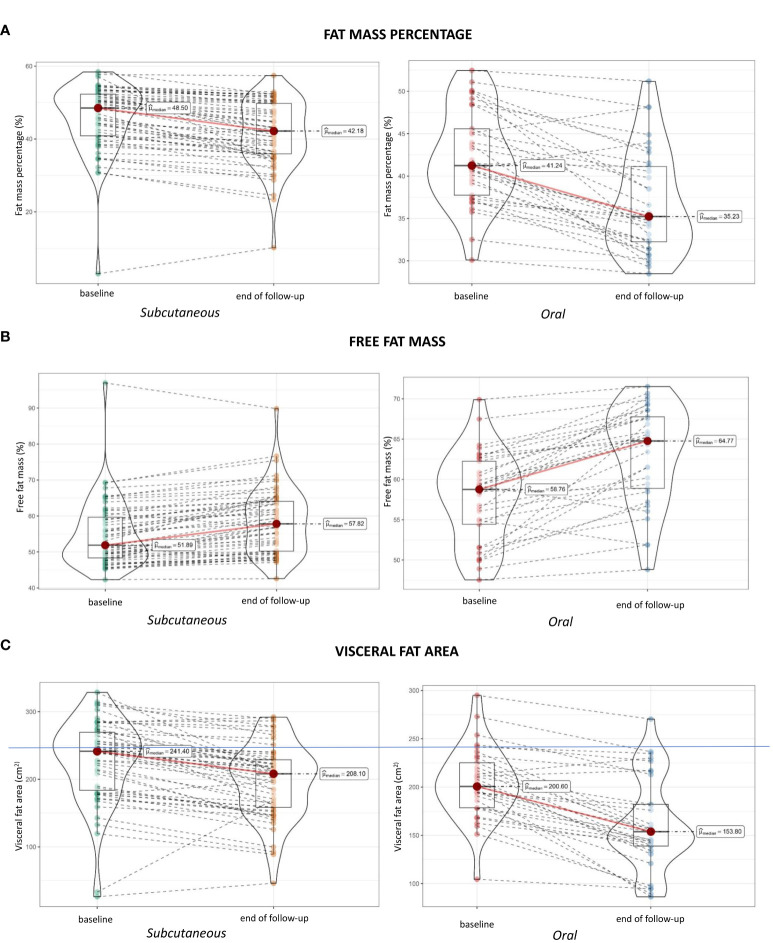
Changes in the composition parameters from baseline to end of follow-up.

Throughout follow-up, participants experimented a significant reduction of their weight, 9.5 ± 5.7% with SS and 9.4 ± 5.9% with OS. We observed that weight loss was at the expense of reducing FM percentage [-4% (IC 95% [-4.9; -3.1], p< 0.001) in SS and -5.2% (IC 95%[-6.7; -3.6], p< 0.001) in OS] while increasing the percentage of FFM [3.6% (IC 95% [2.8; 4.6], p< 0.001) in SS and 5.2% (IC 95%[3.6; 6.7], p< 0.001) in OS] (**Figure 2**).

Considering weight reduction in absolute numbers, in the SS group, loss of FM was -8.5 kg (95% CI [-10.2; -6.9], p < 0.001), while the reduction in lean mass was -1.7 kg (95% CI [-2.4; -1], p < 0.001). These results were consistent in the OS group, where the loss of FM was -8 kg (95% CI [-9.7; -6.2], p < 0.001), and that of lean mass -0.7 kg (95% CI [-1.5; 0.2], p = 0.112).

Nevertheless, PhA remained constant on average during follow-up [-0.03° (CI 95% [-0.1; 0.1], p = 0.529) in SS and -0.1° (CI 95% [-0.2; 0], p = 0.068) in OS].

Changes in other body composition parameters were also seen, such as a decrease in total body water and visceral fat area (VFA), which was -30.2cm^2^ (CI 95% [-37.5; -23.2], p <0.001) in SS and -42.3 cm^2^ (CI 95% [-52.9; -31.8], p <0.001) in OS. **Table 2** complies information regarding these changes.

### Effect on analytical and clinical parameters

In addition to the benefit observed on patients’ body composition, their laboratory metrics also improved during the follow-up period. For instance, lipid profile demonstrated positive changes with a reduction in total cholesterol of -30mg/dl (CI 95% [-44.5; -17.5], p < 0.001) in SS and -49.4mg/dl (CI 95% [-70; -28.8], p < 0.001) in OS (**Table 3**). At the same time, metabolic control was optimized with a reduction in HbA1c -3.8% (CI 95% [-4.5; -3], p < 0.001) in SS and -3% (CI 95% [-3.7; -2.2], p < 0.001) in OS; achieving optimal metabolic control (HbA1c <7%) in 92.3% of patients undergoing treatment with SS and 75.8% in those who received OS. In both groups, we observed a decrease in insulin requirements after 6 months, with statistical significance noted in the SS group, where patients required 4 UI (IC 95% (–9, 0), p =0.01) less.

**Table 3:** Changes in analytical parameters from baseline to end of follow-up (6 months).

	SUBCUTANEOUS SEMAGLUTIDE (n=55)				ORAL SEMAGLUTIDE (n = 33)			
	BASELINE mean (SD)	6 MONTHS mean (SD)	DIFFERENCE [CI 95%]	P-value^a^	BASELINE mean (SD)	6 MONTHS mean (SD)	DIFFERENCE [CI 95%]	P-value^a^
**HbA1C (%)**	9.9 (2.1)	6 (0.7)	-4 [-4.6; -3.3]	<0.001	9.3 (1.9)	6.4 (0.9)	-3 [-3.7; -2.2]	<0.001
**Fasting plasma glucose** **(mg/dl)**	217.5 (75.8)	101.8 (23.8)	-114 [-139; -92.5]	<0.001	190.8 (57.7)	100.2 (25.5)	-90.5 [-118; -66]	<0.001
**TC (mg/dl)**	198.2 (49.6)	162.4 (33.6)	-30 [-44.5; -17.5]	<0.001	194.5 (52.4)	147 (35.8)	-49.4 [-70; -28.8]	<0.001
**HDL cholesterol** **(mg/dl)**	43.3 (12.4)	45.9 (9.6)	2.4 [0; 4.7]	0.054	41.4 (11.9)	42.3 (11.5)	1.6 [07; 3.8]	0.16
**LDL-cholesterol** **(mg/dl)**	110 (46.4)	89.2 (31.1)	-16 [-28.5; -5.5]	0.006	107.9 (43.8)	71.4 (30.8)	-38.1 [-54; -22.1]	<0.001
**non-HDL cholesterol** **(mg/dl)**	150.3 (49.2)	117.6 (30.4)	-25 [-43; -14]	<0.001	48.7 (153.8)	102.8 (33)	-51 [-71.9; -30.1]	<0.001
**Triglycerides (mg/dl)**	314.1 (569)	145.9 (48.8)	-70 [-99.5; -45]	<0.001	253.2 (168.5)	163.1 (70)	-68 [-117; -28.5]	0.001
**Creatinine (mg/dl)**	0.8 (0.2)	0.81 (0.3)	0.02 [-0.02; 0.06]	0.287	0.9 (0.4)	0.9 (0.4)	0.01 [-0.04;0.05]	0.681
**eGFR** **(mL/min/1.73m²)**	95.7 (18.8)	93.4 (19.4)	-1.5 [-4.5; 0.5]	0.119	84.2 (21.1)	83 (21.8)	1 [-2; 3]	0.559
**Uric acid (mg/dl)**	4.87 (1.3)	4.94 (1.6)	0.2 [-0.4; 0.5]	0.35	5.8 (1.8)	3.8 (0.7)	-0.9 [-2; 0.2]	0.07
**Haemoglobin (mg/dl)**	14.7 (1.7)	15.1 (1.8)	0.4 [0.1; 0.7]	0.012	14.4 (1.4)	14.6 (1.7)	-0.2 [-0.8; 0.3]	0.387
**Platelet count** **(x 10^3/Ml)**	314.9 (342.2)	264.4 (67.8)	16.5 [5.5; 28.5]	0.061	258.5 (78.8)	267.6 (88.5)	1.6 [-16.8; 20]	0.862
**AST (U/L)**	37.1 (33.5)	20.1 (7.5)	-10.5 [-19; -4.5]	<0.001	37.2 (28.8)	21.6 (10.4)	-8 [-15; -3.5]	0.003
**ALT (U/L)**	43.6 (39.2)	21.5 (12)	-14.5 [-23; -9]	<0.001	43.7 (29.7)	22.4 (12.8)	-15 [-25; -9.5]	<0.001
**TSH (μUI/mL)**	6.9 (25.5)	2.1 (15)	-0.3 [-0.7; -0.1]	0.005	2.6 (2.4)	1.9 (1.1)	-0.3 [-0.9; 0]	0.056
**FIB-4**	1.2 (0.8)	0.9 (0.4)	-0.2 [-0.3; 0]	0.026	1.6 (1.9)	1.4 (1.5)	0 [-0.6; 0.2]	0.856

^a^ Univariate analysis using Wilcoxon test for paired data.

SD, Standard Deviation; Cl, Confidence Interval; eGFR, Estimated Glomerular Filtrate Rate; TC, Total Cholesterol; HDL, High-Density Lipoprotein Cholesterol; LDL, Low-Density Lipoprotein Cholesterol; Non-HDL, Non-High-density Lipoprotein Cholesterol; AST, Aspartate Aminotransferase; ALT, Alanine Aminotransferase; Hbalc, Giycated Haemoglobin; TSH, Thyroid-Stimulating Hormone; FIB-4, Fibrosis-4 Index for Liver Fibrosis.

### Multivariate analysis

Additionally, we performed a multivariate analysis using multiple linear regression, with the percentage of mass loss at the end of follow-up as the dependent variable for all study participants (n=88). As independent variables, we included the administration route of semaglutide, along with those variables where significant differences were identified at baseline. We observed that percentage of FM lost at the end of follow-up was determined by the initial percentage of FM, baseline BMI and concomitant treatment with iSGLT2 (sodium-glucose linked transporter inhibitors) and insulin. Notably, no significant association was observed with the treatment administration route (**Table 4**).

**Table 4:** Multivariate analysis using multiple linear regression to adjust for predictor variables of fat mass loss percentage.

	**B**	**95% CI**	**p-value**
**Age**	0.014	[-0.03; 0.06]	0.503
**Baseline Fat Mass**	0.11	[0.05; 0.18]	0.001
**Baseline Visceral Fat Area**	-0.05	[-0.18; 0.05]	0.275
**Subcutaneous/Oral Semaglutide**	-0.563	[-1.4; 0.281]	0.188
**iSGLT2 treatment**	1.01	[0.21; 1.81]	0.014
**Insulin treatment**	0.95	[0.18; 1.71]	0.017

Dependent variable: Fat mass loss percentage.

Statistics: R2 0.468; p < 0.001.

BMI, Body Mass Index; iSGLT2, sodium-glucose linked transporter inhibitors; 95% CI, 95% Confidence Interval.

## Discussion

T2DM is a highly prevalent disease accounting for nearly 95% of all diabetes (24), with a rapidly increasing prevalence. Noticeably, a substantial proportion of individuals with T2DM remain unaware of their diagnosis, leading to lost opportunities in early management. Meanwhile, the global prevalence of obesity has almost tripled since 1975 according to the World Health Organization. We conducted a study that examines the changes observed in an adult population with T2DM and obesity, focusing on modifications in body composition evaluated through MF-BIA.

Assessing body composition is valuable for individual evaluation and estimating changes over time. Beyond anthropometry, MF-BIA provides insights into various body components and their interrelations in a given person (25). Other methods used to study body composition include dual-energy x-ray absorptiometry (DXA) and magnetic resonance imaging (MRI) among others. DXA offers accurate measurements of various body components in addition to bone mineral density data. Conducted with standardized protocols, these measurements ensure consistency and comparability across diverse studies and populations. Several studies have indicated comparable outcomes between DXA and MF-BIA (26–29). For instance, Schoenfeld et al. found no significant differences in mean changes between MF-BIA and DXA scans for FM, percent body fat, and FFM (30). Similarly, other investigations comparing MF-BIA with DXA in body composition evaluation reported similar findings for total body and segmental soft tissue measures (31). Likewise, MRI’s precision allows detailed visualization of various tissues, aiding accurate assessment of fat distribution, muscle mass, and other components. MRI’s whole-body imaging capability enables comprehensive evaluation of adipose tissue and lean mass distribution, offering valuable insights into overall health. It is non-invasive, ensuring patient safety with high-resolution images and no ionizing radiation. MRI’s quantitative analysis allows precise measurements of tissue volumes and densities, facilitating quantitative assessments of fat content and distribution. This makes it valuable for diagnosing and monitoring conditions like obesity, sarcopenia, and metabolic disorders. Its wide acceptance among patients and versatility in research further highlight MRI’s significance for assessing body composition and guiding clinical decisions in medical settings (32).

In contrast, BIA offers several advantages for assessing body composition in medical practice. Its non-invasive nature ensures patient comfort and safety, being suitable for individuals of different ages and health conditions. BIA devices are portable, affordable, and easy to use, making them accessible in various healthcare settings. The rapidity of BIA assessments allows for efficient incorporation into clinical practice, providing quick insights into body composition without significant time investment. Additionally, BIA’s versatility permits detailed assessments of both total and segmental body composition, offering valuable information on fat and lean mass distribution. Longitudinal monitoring capabilities enable clinicians to track changes in body composition over time, aiding in the management of conditions such as obesity or muscle wasting disorders (25).

BIA estimates body composition based on a series of predictive equations developed for a specific population. Alongside data on the different body compartments, BIA provides raw electrical values: impedance, resistance, reactance and PhA (the arctangent of the ratio of reactance to resistance (33)). BIA calculates secondary data on fluid compartments, such as total body water, intracellular water, and extracellular water. From these, fat-related compartments (FM and FFM) are derived, as the body’s water content primarily resides in FFM (34). Consequently, achieving optimal outcomes for entire body compartments, even in individuals with good health, relies on choosing a suitable equation (35). Regardless of its inherent limitations as an indirect method, BIA continues to be extensively used in both epidemiological studies and individual assessments.

Regarding body composition, while the baseline characteristic of the groups differed, our multivariate model revealed a similar impact of oral versus subcutaneous semaglutide on weight loss. This suggests that there may not be significant differences between the two administration forms. Nonetheless, it is crucial to conduct studies with a specific design to thoroughly evaluate this hypothesis. Our cohort experimented a reduction in their body weight of -10kg (-9.5%) and -8.6kg (-9.4%) depending on semaglutide’s route of administration (SS and OS respectively), slightly higher than that described in literature (23). Weight reductions ranging from 5–10% are considered clinically significant and correlate with cardiovascular risk improvement (36). We observed that weight loss was to a greater extent due to a reduction in the percentage of FM than the percentage of FFM, suggesting a decrease of FM without significant losses in the lean mass component.

There has been ongoing discourse in the literature regarding a potential association between Semaglutide use and the onset of sarcopenia. However, most studies indicate that Semaglutide primarily induces weight loss by reducing FM while either preserving muscle mass or even enhancing the relative proportion of skeletal muscle, with minimal or non-clinically relevant impact on muscle strength (37–40). In our cohort, we observed that the loss of muscle tissue was not significant in the OS group. Conversely, in the SS group, there was a statistically significant reduction in muscle tissue, which could be attributed to the greater overall weight loss compared to the OS group. Given that individuals starting the SS option had, on average, a higher BMI and thus a greater degree of obesity, they also began with a higher baseline lean mass compared to those in the OS group, which could explain why they experienced a proportionally greater decrease in their lean mass component. The Skeletal Muscle Index (kg/m2) decreased in both groups, with significant reductions in absolute values observed only in the SS group. These findings underscore the apparent predominantly positive impact of semaglutide on fat mass reduction. However, considering our results and previous research (20), it would seem highly advisable to implement targeted physical exercise to preserve and potentially enhance muscle mass in patients receiving GLP-1 based therapies.

Traditionally, the association between BMI and long-term all-cause mortality was described as a U-shape (41), as a result of different underlying risk functions of opposite directions associated with fat and fat-free body compartments (42). Recent investigation has shown an inverse association between FFM and mortality, whereas FM was directly associated with mortality (43). A clinical study including a series of patients with BIA assessment before and after follow-up, showed increased mortality by weight loss probably attributed to loss in FFM (44). Likewise, another study suggested that the increase in mortality seen in their population was less in subjects who alongside with their weight loss has a relatively high physical activity (45). This highlights how detrimental the FM component is and points out the need to consider it in clinical practice, emphasising that relying solely on BMI could be insufficient when evaluating a patient’s metabolic state.

Our cohort underwent a reduction in VFA after treatment with semaglutide both in SS and OS routes of administration. VFA plays a key role in metabolic and cardiovascular disease. Visceral adipose tissue secretes inflammatory mediators leading to a chronic low grade inflammation state (46, 47), which induces a constellation of metabolic abnormalities and diseases (46). Adiposity is an independent risk factor for glycometabolism disorder, since fat accumulation affects insulin’s action by different mechanisms including insulin resistance in organs such as the liver and muscle tissue (46, 48). Over the years, evidence has suggested that the risk of adiposity-induced metabolic dysfunction is not solely determined by the quantity of accumulated fat in the body, but rather by its distribution. According to Klein et al., decreasing body FM by inducing a negative energy balance could normalize obesity-induced dysfunction.

Focusing on PhA, throughout follow-up it maintained similar values as those registered in the first body composition analysis. From these results we can infer that Semaglutide did not affect cellular integrity to a significant extent. PhA acts as a marker of the amount of electrical charge that a cell membrane can hold, related to membrane integrity, permeability, overall size and hydration (35); therefore, indicating cellular health and function (49). PhA is a raw bioelectrical parameter with a demonstrated prognostic utility (50). Its value is influenced by various factors: aging contributes to a decline on PhA as muscle mass decreases, men typically exhibit higher PhA values than women owing to greater muscle mass, and there is a positive correlation between BMI and PhA, with higher BMI values corresponding to increased PhA values. Several studies have demonstrated lower values of PhA in T2DM, relating smaller values with catabolism and longer disease duration (49). The main challenge of using PhA in clinical evaluation is the lack of consensus on cut-points and reference values. Several investigations have been carried out around the world, based on large population samples in Germany (51), the United States (52) and Switzerland (53), obtaining notable differences (35). Therefore, further research is required to establish, through standardized measurement techniques, reference cut-off points (54).

With respect to analytical parameters, glycaemic control improved significantly as proven by HbA1c and fasting plasma glucose. Clinical trials have reported reductions in the HbA1c level to be as high as 1.8%, depending on dosage and follow-up duration (23). This percentage is lower than our findings, which could correspond to higher HbA1c levels at baseline in our cohort compared to clinical trials, where inclusion criteria are more stringent, since our study was conducted in a real-life clinical practice setting. Attaining desired blood glucose levels is a key objective in diabetes management, since optimal glycaemic control reduces the risk of both microvascular and macrovascular complications (36, 55). In this regard, as part of everyday clinical practice, an educational activity aimed at reinforcing healthy lifestyle habits was carried out simultaneously with the treatment, which could have influenced a better weight response and glycaemic control. This protocol is standard within our endocrinology service and is routinely practiced, both within the scope of this study and as part of routine clinical care. Therefore, no assessment of its impact was conducted. GLP-1ra enhance glycaemic control by stimulating insulin secretion and by a glucose-dependent inhibition of glucagon release (56), resulting in effective glucose lowering without increased risk of hypoglycaemia (57). Consistent with this knowledge, glycaemic improvement in our cohort was not accompanied by more frequent hypoglycaemic events.

Additionally, basal insulin requirements in our cohort decreased after follow-up, highlighting the fact that our patients were metabolically in better conditions after semaglutide treatment. This is a promising finding, since insulin, although effective in hyperglycaemic control for many patients, is associated with hypoglycaemia and weight gain (57).

Lipid profile showed improvement as well, with significant reductions in total-cholesterol and non-significant reduction in Low-density lipoprotein cholesterol concentrations. This is consistent with SUSTAIN 9’s trial, where both parameters significantly decreased in the same proportion (58). It is suggested that such improvement could come alongside with reductions in body weight and glucose levels; GLP-1ra have also been shown to regulate cholesterol and triglyceride concentrations via several different pathways (59).

Recently, two studies similar to ours have been published, yet with certain differences. Both studies investigated changes in body composition after 26 weeks of treatment with semaglutide. One study (60), assessed the impact of OS in 32 patients with a mean BMI of 28.2 kg/m2. Results showed a weight loss of 4 kg at 6 months, accompanied by improvements in lipid profiles and a significant reduction in HbA1c at 3 and 6 months of follow-up. In the other study (37), involving subcutaneous administration of semaglutide in a population of 40 patients with a mean BMI of 38.8 kg/m2, a weight loss of 9.89kg was observed after 6 months. This second study showed a weight loss pattern consistent with our findings, probably influenced by a similar baseline BMI between the two studies. It is noteworthy that both studies established as an exclusion criterion the concomitant use of other hypoglycaemic agents such as iSGLT2 inhibitors. Additionally, not all patients reached full semaglutide dose: in the SS study, only 5% reached a weekly dose of 1 mg; in the OS study, all patients remained on an intermediate (7 mg) or initial dose (3 mg). In other words, the patients did not uniformly receive the same treatment regimen. Furthermore, sample size in both studies was limited.

Previous investigations have delved into the impact of combining physical activity programs with nutritional recommendations to aid weight reduction. It is widely recognized that structured lifestyle interventions play a pivotal role in facilitating weight loss among individuals grappling with obesity (61). Such interventions can yield significant outcomes, with a 3–5% reduction in body weight achievable through health behavioural changes, leading to tangible improvements in obesity-related comorbidities (62). Notably, Wilding et al. highlighted that combining Semaglutide treatment with lifestyle interventions effectively sustained clinically relevant weight loss in overweight or obese adults (1). In our study, patients were provided with general recommendations for adopting a healthy lifestyle and engaging in physical exercise, cantered around the Mediterranean diet and active living to deter sedentary behaviour. These guidelines were initially communicated during the first visit and subsequently reinforced by our nursing team during a brief 15-minute session. While this information was uniformly disseminated to all participants, there was no subsequent follow-up or utilization of specific programs for reinforcement.

## Limitations

We acknowledge some limitations in our study. We did not investigate a control group with which to compare the effects of the treatment on the different studied parameters. The duration of the study was limited; longer-term data would have provided more information and potentially greater modifications in body composition as well as analytical parameters. Another limitation was narrow sample size, which although proved to be statistically adequate, did not allow for a more in-depth analysis of the studied variables. Furthermore, the retrospective nature of the study represents a weakness, as well as not being able to compare our treatment groups due to the basal differences observed between them. Additionally, BIA is not considered the gold standard method, and we recognize that incorporating other body composition tests may be necessary in future research.

On the other hand, it is clearly a strength that body composition parameters were estimated from BIA rather than anthropometric predictions. Likewise, it is an advantage that our data was recorded on a real-life basis. This enhances the generalizability of our findings, given that real-world studies include a more diverse and representative patient population compared to clinical trials. Consequently, our results hold greater applicability across a broader range of individuals. Additionally, such studies directly influence clinical practice, guiding healthcare providers in making evidence-based decisions in their daily patient care.

## Conclusion

In light of these results, we conclude that semaglutide improves body composition parameters with significant losses of body FM with a lesser compromise of lean mass, as well as positively influencing glycaemic control and metabolic status. The potential for muscle mass decline exists; therefore, it should be anticipated and addressed through regular physical activity to mitigate it, considering that although this decrease is expected to be less pronounced than that of fat mass, it remains likely to occur. Integrating BIA into clinical practice enables the evaluation of antidiabetic drugs’ impact on the human body from a new perspective, paving the way for upcoming studies.

## Data availability statement

The original contributions presented in the study are included in the article/supplementary material. Further inquiries can be directed to the corresponding author.

## Ethics statement

The studies involving humans were approved by Research Ethics Committee of the Hospital Universitario Virgen Macarena of Seville. The studies were conducted in accordance with the local legislation and institutional requirements. The participants provided their written informed consent to participate in this study.

## Author contributions

BJ: Writing – review & editing, Writing – original draft, Visualization, Supervision, Project administration, Methodology, Investigation, Formal analysis, Data curation, Conceptualization. PG: Writing – review & editing, Writing – original draft, Visualization, Validation, Supervision, Project administration, Methodology, Investigation, Formal analysis, Data curation, Conceptualization. SL: Writing – original draft, Data curation. ÁD: Writing – original draft, Data curation. IM: Writing – original draft, Investigation, Data curation. IG: Writing – original draft, Investigation, Data curation. CP: Writing – review & editing, Resources, Investigation, Conceptualization. MM-B: Writing – review & editing, Visualization, Supervision, Project administration.
